# Alfalfa xeno-miR159a regulates bovine mammary epithelial cell proliferation and milk protein synthesis by targeting PTPRF

**DOI:** 10.1038/s41598-024-59948-x

**Published:** 2024-04-20

**Authors:** Hongjuan Duan, Shaojin Li, Chuangwei Li, Lan Tian, Yun Ma, Xiaoyan Cai

**Affiliations:** Key Laboratory of Ruminant Molecular and Cellular Breeding of Ningxia Hui Autonomous Region, College of Animal Science and Technology, Ningxia University, Yinchuan, 750021 China

**Keywords:** Cell biology, Microbiology, Molecular biology, Non-coding RNAs, RNAi

## Abstract

Milk protein content is an important index to evaluate the quality and nutrition of milk. Accumulating evidence suggests that microRNAs (miRNAs) play important roles in bovine lactation, but little is known regarding the cross-kingdom regulatory roles of plant-derived exogenous miRNAs (xeno-miRNAs) in milk protein synthesis, particularly the underlying molecular mechanisms. The purpose of this study was to explore the regulatory mechanism of alfalfa-derived xeno-miRNAs on proliferation and milk protein synthesis in bovine mammary epithelial cells (BMECs). Our previous study showed that alfalfa miR159a (mtr-miR159a, xeno-miR159a) was highly expressed in alfalfa, and the abundance of mtr-miR159a was significantly lower in serum and whey from high-protein-milk dairy cows compared with low-protein-milk dairy cows. In this study, mRNA expression was detected by real-time quantitative PCR (qRT-PCR), and casein content was evaluated by enzyme-linked immunosorbent assay (ELISA). Cell proliferation and apoptosis were detected using the cell counting kit 8 (CCK-8) assay, 5-ethynyl-2′-deoxyuridine (EdU) staining, western blot, and flow cytometry. A dual-luciferase reporter assay was used to determine the regulation of Protein Tyrosine Phosphatase Receptor Type F (PTPRF) by xeno-miR159a. We found that xeno-miR159a overexpression inhibited proliferation of BMEC and promoted cell apoptosis. Besides, xeno-miR159a overexpression decreased β-casein abundance, and increased α-casein and κ-casein abundance in BMECs. Dual-luciferase reporter assay result confirmed that PTPRF is a target gene of xeno-miR159a. These results provide new insights into the mechanism by which alfalfa-derived miRNAs regulate BMECs proliferation and milk protein synthesis.

## Introduction

Dairy milk is generally considered as an important source of protein in the human diet. Milk protein is the main nutritional component of milk and an important nutritional indicator of milk quality. Dairy cow milk proteins mainly include casein (including αs1-casein, αs2-casein, β-casein, and κ-casein,), which accounts for approximately 80% of milk protein, and whey proteins (including α-lactalbumin and β-lactoglobulin), which account for approximately 20%^1,2^. Therefore, understanding the molecular mechanisms by which diet can influence casein synthesis is essential for optimizing the nutritional content of milk from ruminant animals, as well as for improving the milk protein content of dairy products.

MicroRNAs (miRNAs) are non-coding single-stranded RNAs with a length of about 20–25 nucleotides, which are abundant in eukaryotic cells. Interestingly, recent studies suggest that plant-derived miRNAs, so-called exogenous miRNAs (xeno-miRNAs), can have cross-kingdom functions. For example, miR-168a from rice was shown to regulate mammalian gene expression after dietary intake^3,4^. However, research on the regulatory roles of plant-derived miRNAs are mainly focused on tumor prevention and treatment, the effects of plant-derived miRNAs on dairy milk casein synthesis remains unknown, needs to be learned further.

Alfalfa is an important dietary component to guarantee high protein content in milk from dairy cows. Alfalfa miRNA (mtr-miRNA) has been shown to have cross-kingdom regulatory functions. Plant mtr-miR5754 was found to directly target the tumor-associated long non-coding RNA metastasis-associated lung adenocarcinoma transcript 1 (MALAT1) in a sequence-specific manner, to reduce the stability of these oncogenic target transcripts, and to inhibit cancer cell proliferation^5^. In addition, our research group showed that over-expression of alfalfa xeno-miR168b inhibits bovine mammary epithelial cell (BMEC) proliferation and reduces lipid droplets and triglyceride content in BMECs^6^. An earlier study found that oral administration of a plant miR159 mimic significantly suppressed the growth of xenograft breast tumors in mice, indicating that dietary plant-derived miR159 may be able to influence breast cancer growth^7^, and demonstrating for the first time that a plant miRNA can inhibit cancer growth in mammals. However, whether plant xeno-miR159a plays a role in the synthesis and secretion of dairy milk protein has not been determined. Based on RNA-seq previously conducted by our research group showed that mtr-miR159a was highly expressed in alfalfa, we hypothesized that alfalfa xeno-miR159 (mtr-miR159a) may play a role in improving milk protein content. Our results will lay a foundation for further analysis of xeno-miRNA regulation of the molecular networks controlling BMEC proliferation and milk protein synthesis.

## Results

### Alfalfa miRNA screening

**Figure 1:**
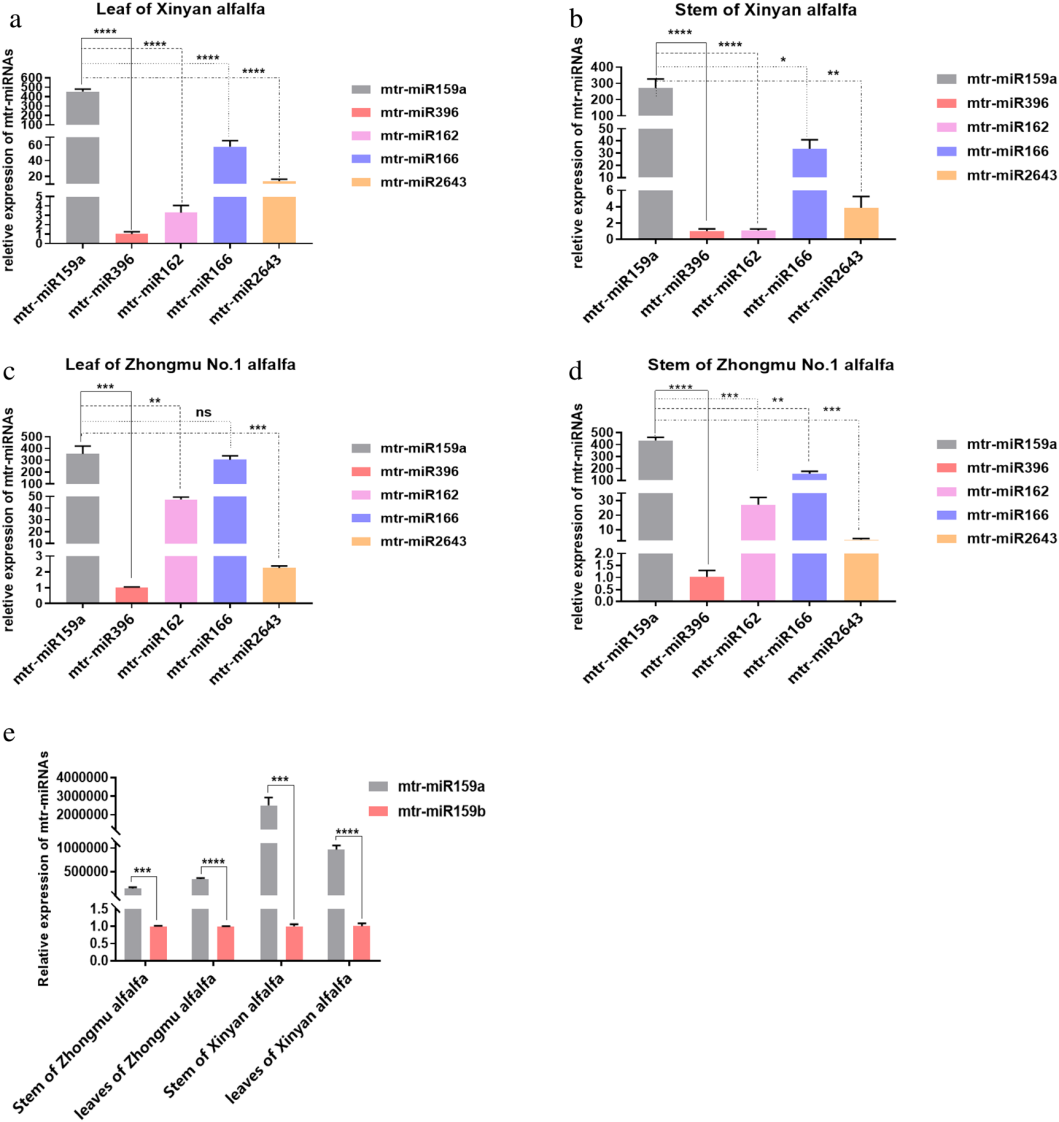
Analysis of miRNA expression in stem and leaves of two alfalfa varieties. (**a**–**d**) qRT-PCR analysis of indicated miRNAs in (**a**) leaf of Xinyan alfalfa, (**b**) stem of Xinyan alfalfa, (**c**) leaf of Zhongmu No. 1 alfalfa, and (**d**) stem of Zhongmu No. 1 alfalfa. (**e**) qRT-PCR analysis of mtr-miR159a and mtr-miR159b expression in stem and leaves of Xinyan and Zhongmu No. 1 alfalfa. *p < 0.05; **p < 0.01; ***p < 0.001; ****p < 0.0001; ns indicates p > 0.05.

We previously reported RNA-seq data indicating that mtr-miR159a, mtr-miR396a, mtr-miR166a, mtr-miR2643a, and mtr-miR162 are highly expressed miRNAs in two alfalfa varieties (Zhongmu No.1 alfalfa and Xinyan alfalfa) (Table S2), and that, among these, mtr-miR159a was the most highly expressed^6^. To validate the RNA-seq results, here we measured miRNA expression in alfalfa extracts using qRT-PCR. Consistent with our previous findings, mtr-miR159a was the mostly highly expressed of the five miRNAs in both stem and leaf of two alfalfa varieties (Fig. 1a–d). There are 5 members of mtr-miR159, including mtr-miR159a, mtr-miR159b, mtr-miR159c, mtr-miR159d, mtr-miR159e, among them, mtr-miR159a, d, and e share a same mature sequence, while mtr-miR159b and mtr-miR159c have same mature sequence. Thus, mtr-miR159a and mtr-miR159b were selected to perform qRT-PCR to compare the expression of mtr-miR159a with mtr-miR159b in stem and leaves of the two alfalfa varieties, and we found that mtr-miR159a expression was significantly higher than mtr-miR159b in both varieties and in both tissues (Fig. 1e). Based on these data, in addition to direct demonstration of cross-kingdom regulatory effects of miR159a^5^, mtr-miR159a was prioritized for the subsequent experiments.

### Alfalfa xeno-miR159a in dairy cow milk and blood

**Figure 2:**
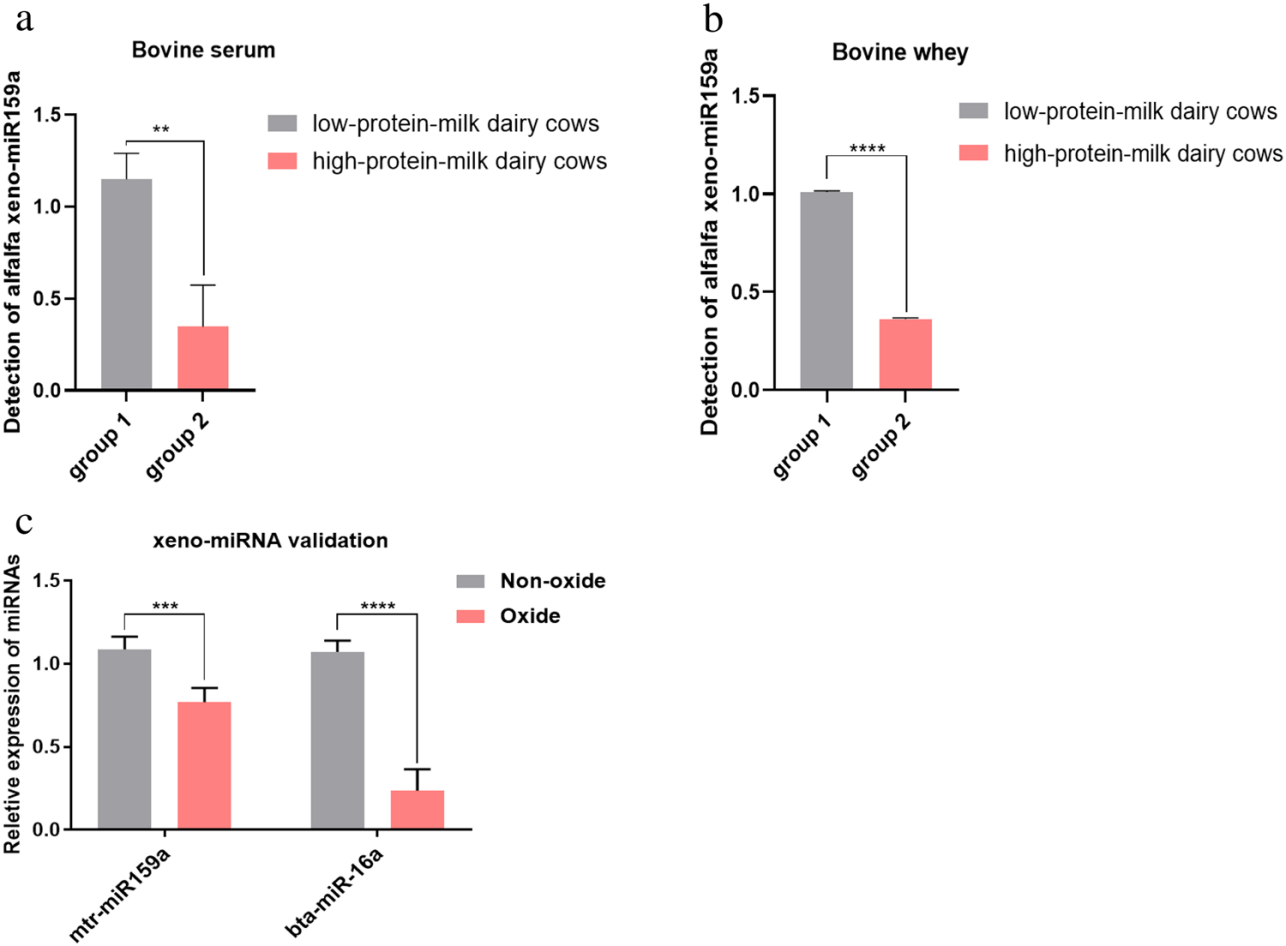
Detection of alfalfa xeno-miR159a in dairy cow milk and blood. (**a**,**b**) Levels of mtr-miR159a in low-protein-milk dairy cows and high-protein-milk dairy cows in (**a**) serum and (**b**) whey. (**c**) Levels of mtr-miR159a and endogenous bta-miR-16a in bovine blood before and after oxidation. *p < 0.05; **p < 0.01; ***p < 0.001; ****p < 0.0001.

Because mtr-miR159a was highly expressed in alfalfa, we measured the abundance of alfalfa xeno-miR159a in the serum and whey of dairy cows, and we evaluated whether the abundance levels of alfalfa xeno-miR159a differed between dairy cows with high-protein (average 3.5 ± 0.54, > 3.3%) versus low-protein milk (average 2.7 ± 0.13, < 3.0%) by using qRT-PCR. The level of alfalfa xeno-miR159a was significantly lower in high-protein-milk dairy cows compared with low-protein-milk dairy cows in serum (Fig. 2a) and in whey (Fig. 2b). Plant miRNA is resistant to oxidation due to intrinsic differences in methylation patterns of the 3’UTR region, which in turn provides protection from exonucleases and in this case, the oxidizing agent sodium periodate^2^. Thus, an oxidation test was performed to confirm that the detectable xeno-miR159a in cattle serum was indeed of plant origin, which showed that the abundance of plant-derived xeno-miR159a in oxidized bovine blood was decreased by 32% compared with non-oxidized bovine blood, and the expression of endogenous bta-miR-16a in oxidized bovine blood was dereased by 83% compared with non-oxidized bovine blood(Fig. 2c), indicating that alfalfa-derived xeno-miR159a in bovine blood was indeed of plant origin.

### Xeno-miR159a inhibits BMEC proliferation and promotes apoptosis

**Figure 3:**
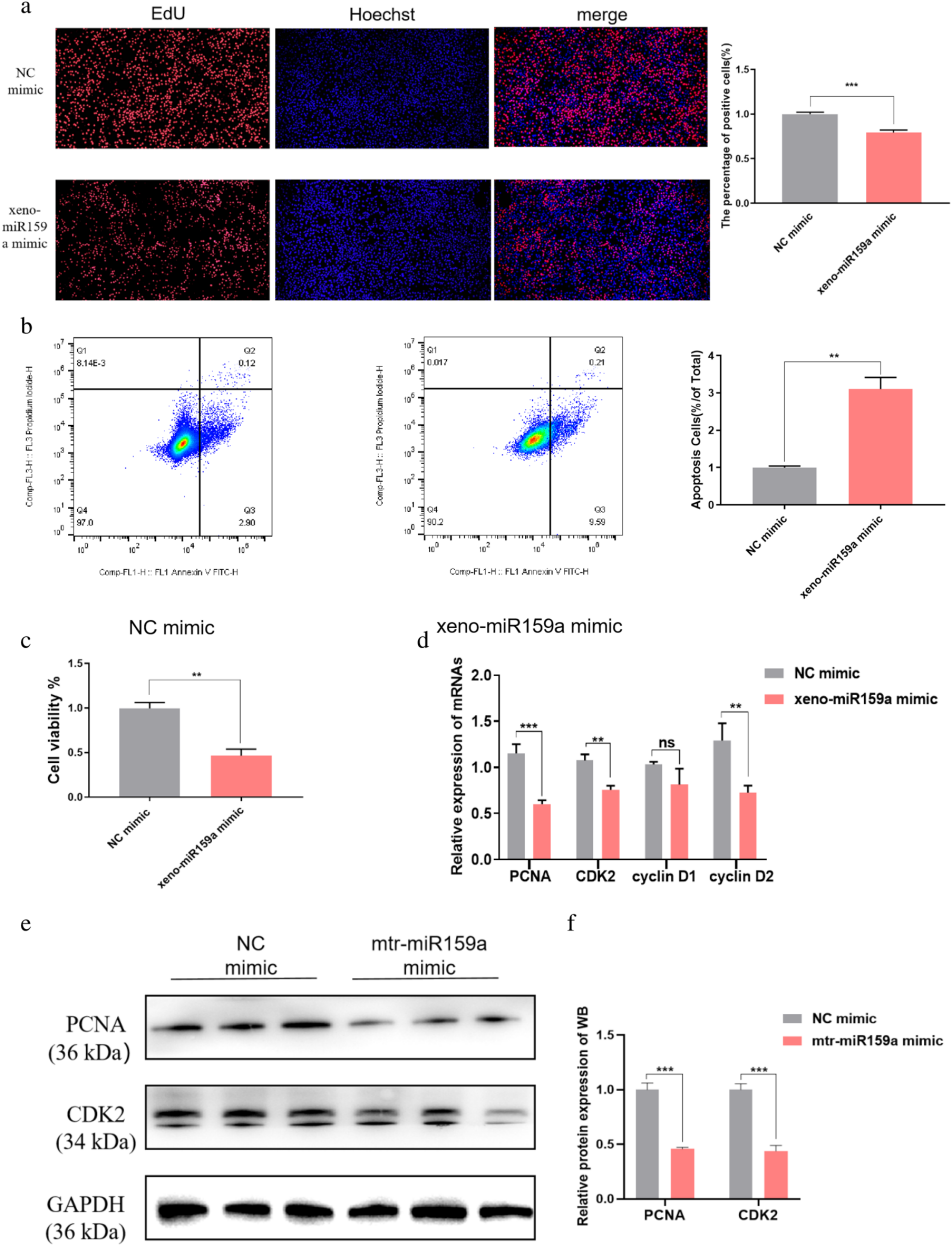
Effect of xeno-miR159a mimic over-expression on BMEC proliferation. (**a**–**f**) BMECs were transfected with xeno-miR159a mimic or NC mimic and assayed after 48 h to detect (**a**) cell proliferation by EdU incorporation, (**b**) Annexin V-FITC apoptosis detection by flow cytometry, (**c**) viability by CCK-8 assay, (**d**) expression of indicated cell proliferation genes by qRT-PCR, and (**e**,**f**) protein levels of proliferation markers by western blot. The samples derive from the same experiment and that blots were processed in parallel; original blots are presented in Supplementary Figure S1. *p < 0.05; **p < 0.01; ***p < 0.001; ns indicates p > 0.05.

To determine whether alfalfa miRNAs have biological effects in dairy cows, we transfected BMECs with a xeno-miR159a mimic or a chemically synthetic miRNA negative control (NC; hereafter, NC mimic) and then assessed proliferation, apoptosis, and viability after 48 h. We found that the xeno-miR159a mimic significantly inhibited cell proliferation compared with NC mimic (Fig. 3a). Furthermore, the xeno-miR159a mimic significantly increased the number of cells in early and late apoptosis compared with controls (Fig. 3b), and the CCK-8 assay showed that xeno-miR159a mimic also significantly reduced cell viability (Fig. 3c). Consistently, qRT-PCR analysis at the same time point showed that xeno-miR159a-mimic-transfected BMECs had significantly down-regulated expression of the proliferation marker genes PCNA, CDK2, Cyclin D1, and Cyclin D2 compared with controls (Fig. 3d). We confirmed the qRT-PCR results by western blot, which showed that, compared with NC mimic, xeno-miR159a mimic significantly decreased protein abundance of PCNA and CDK2 (Fig. 3e, f).

### Xeno-miR159a affects milk protein synthesis by altering mTOR pathway-related gene expression in BMECs

**Figure 4:**
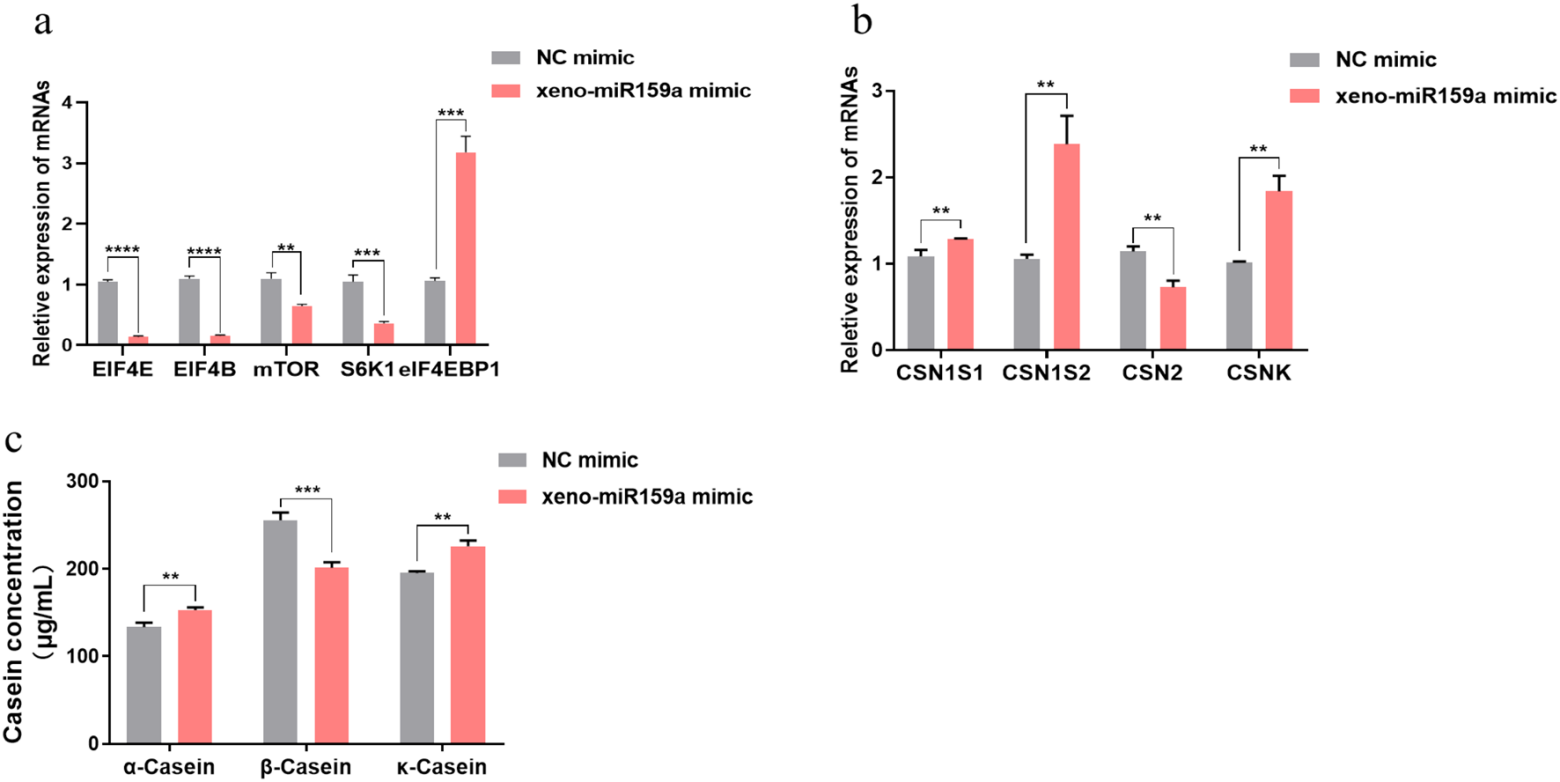
Effect of xeno-miR159a on milk protein synthesis in BMECs. (**a**–**c**) BMECs were transfected with xeno-miR159a mimic or NC mimic and assayed after 48 h to detect (**a**) relative expression of indicated genes in the mTOR signaling pathway by qRT-PCR., (**b**) relative expression of indicated casein-encoding genes by qRT-PCR, and (**c**) casein content in cell supernatant by ELISA *p < 0.05; **p < 0.01; ***p < 0.001; ****p < 0.0001.

The mTOR signaling pathway has been implicated as one of the important regulatory pathways in milk protein synthesis^8^. qRT-PCR analysis was used to evaluated the effect of xeno-miR159a on genes related to the mTOR signaling pathway, including EIF4E, EIF4B, mTOR, S6K1, and eIF4EBP1. Results showed that xeno-miR159a mimic overexpression significantly down-regulated EIF4E, EIF4B, mTOR and S6K1 expression and significantly up-regulated eIF4EBP1 expression in BMECs (Fig. 4a), suggesting that alfalfa xeno-miR159a may affect milk protein synthesis by altering mTOR pathway-related gene expression in BMECs. Next, we evaluated the effect of xeno-miR159a on the expression of the casein-encoding genes CSN1S1, CSN1S2, CSN2, and CSNK using BMECs over-expressing xeno-miR159a mimic or NC mimic. qRT-PCR showed that xeno-miR159a mimic significantly upregulated the expression of CSN1S1 (encoding αS1-casein), CSN1S2 (encoding αS2-casein), and CSNK (encoding κ-casein), but significantly downregulated expression of CSN2 (encoding β-casein) compared with NC mimic (Fig. 4b). To examine whether these changes in gene expression translated to casein content, we then performed ELISA on culture supernatant from BMECs. Compared with the control group, the α-casein and κ-casein levels in supernatant from xeno-miR159a-mimic-transfected BMECs were significantly increased by 14% and 16%, and the β-casein level was significantly decreased by 21% (Fig. 4c). These findings were consistent with mRNA expression levels, indicating that alfalfa xeno-miR159a promotes the synthesis of α-casein and κ-casein and inhibits β-casein synthesis in BMECs.

### Xeno-miR159a targets PTPRF

**Figure 5:**
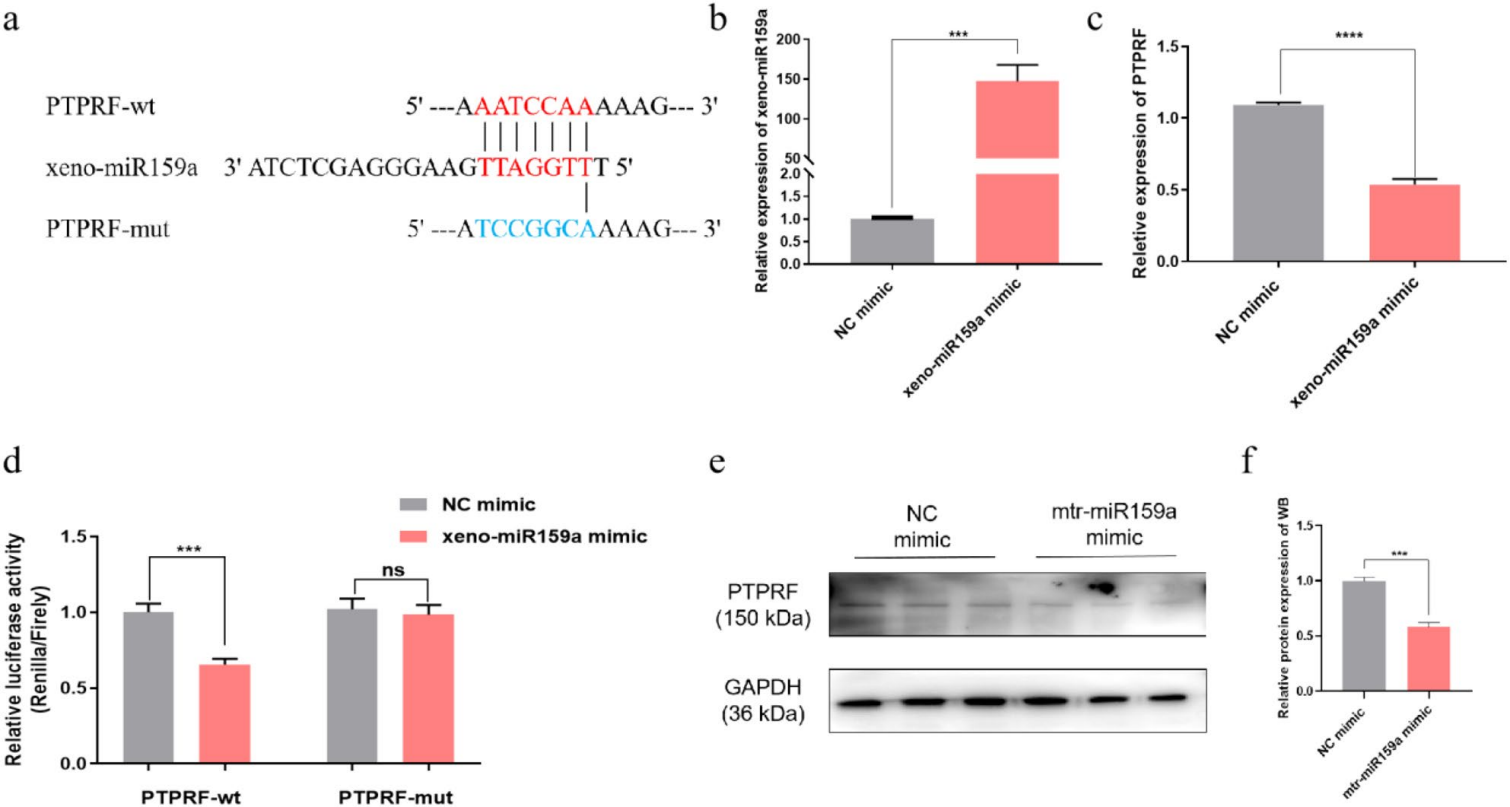
Regulation of PTPRF by xeno-miR159a. (**a**) Alignment of binding sites of alfalfa xeno-miR159a on PTPRF. (**b**) Transfection efficiency of xeno-miR159a mimic in HEK-293 T cells measured by qRT-PCR. (**c**) PTPRF expression in HEK-293 T cells transfected with xeno-miR159a mimic or NC mimic. (**d**) Dual-luciferase reporter assay in HEK-293 T cells transfected with PTPRF-wt or PTPRF-mut vectors and with overexpression of xeno-miR159a mimic or NC mimic. (**e**,**f**) PTPRF protein western blot analysis (**e**) and quantification (**f**) in HEK-293 T cells overexpressing xeno-miR159a mimic or NC mimic. The samples derive from the same experiment and that blots were processed in parallel; original blots are presented in Supplementary Figure S1. *p < 0.05; **p < 0.01; ***p < 0.001; ****p < 0.0001; ns indicates p > 0.05.

To explore the mechanism by which xeno-miR159a may affect milk protein production, we used the bioinformatic tools TargetScan, miRanda, and RNAhybrid to predict binding sites for xeno-miR159a in the bovine genome. Our analysis predicted that xeno-miR159a could bind the 3’UTR of PTPRF; therefore, we cloned wild type (wt) or mutant type (mut) 3′ UTR sequences of PTPRF (Fig. 5a) into the psiCHECK-2 dual luciferase reporter vector, and we co-transfected one of these constructs with miR159a mimic or with NC mimic in HEK-293 T cells. We confirmed that the xeno-miR159a and NC mimics were efficiently expressed in this system (Fig. 5b). PTPRF expression was significantly decreased in miR159a-transfected cells compared with controls (Fig. 5c). Further, in cells transfected with the wild type PTPRF vector, xeno-miR159a mimic significantly reduced luciferase activity compared with NC mimic, but no significant difference in relative luciferase activity was observed between the xeno-miR159a mimic and NC mimic in cells harboring the mutant PTPRF vector (Fig. 5d). Accordingly, over-expression of the xeno-miR159a mimic significantly decreased the abundance of PTPRF at the protein level (Fig. 5e, f). These results demonstrate that xeno-miR159a can regulate PTPRF gene expression.

### PTPRF silencing inhibits BMEC proliferation and promotes apoptosis

**Figure 6:**
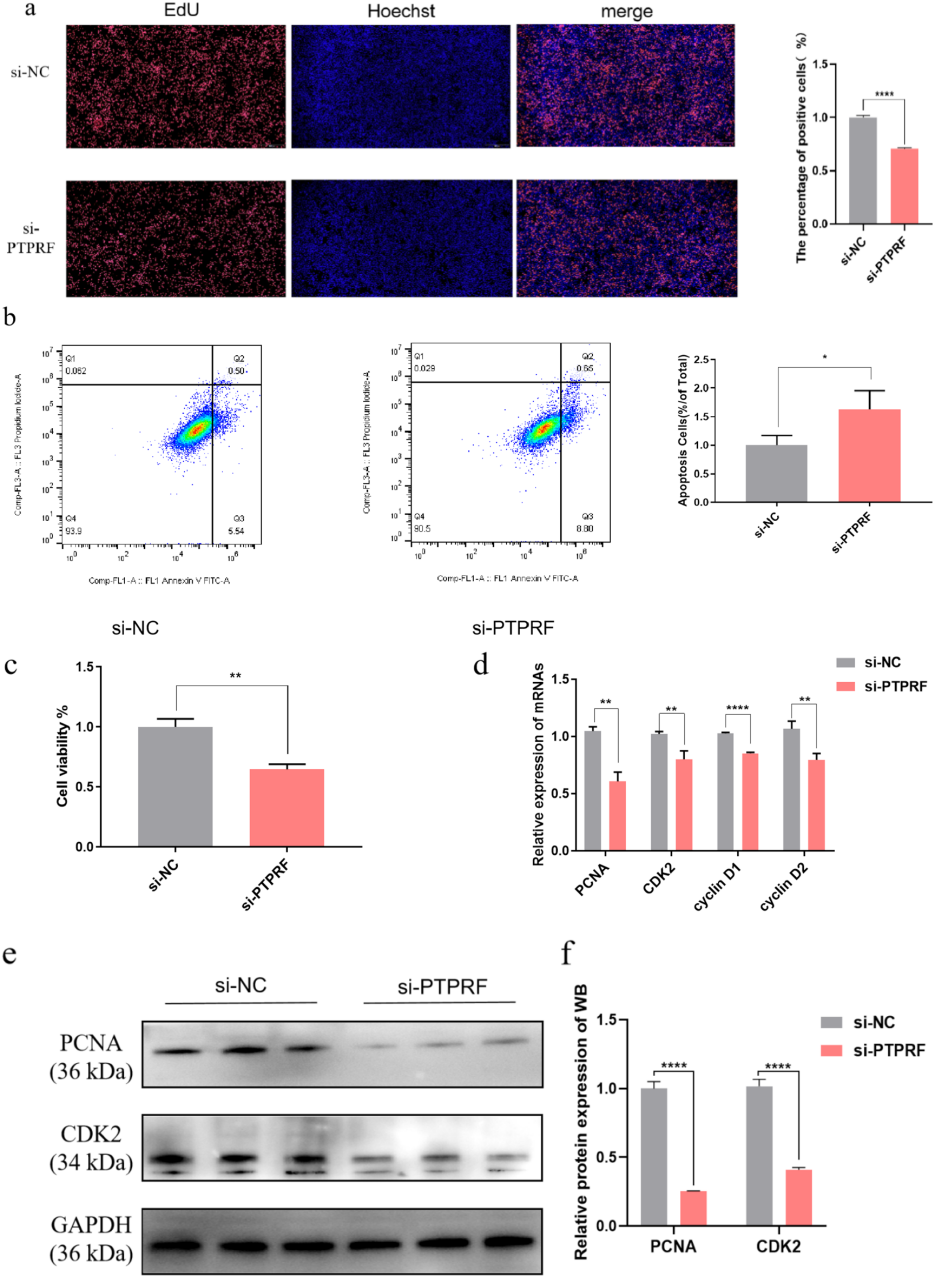
Effect of PTPRF silencing on BMEC proliferation. (**a**–**f**) BMECs were transfected with si-PTPRF or si-NC and assayed after 48 h to detect (**a**) cell proliferation by EdU uptake, (**b**) apoptosis by flow cytometry, (**c**) cell viability using the CCK-8 assay, and (**d**) relative mRNA and (**e**,**f**) protein expression of indicated cell proliferation genes. The samples derive from the same experiment and that blots were processed in parallel; original blots are presented in Supplementary Figure S1. *p < 0.05; **p < 0.01; ***p < 0.001; ****p < 0.0001.

To study the effect of PTPRF on the proliferation, BMECs were transfected with si-NC and si-PTPRF upon reaching approximately 70% confluence, and cell proliferation was measured by EdU uptake after 48 h. PTPRF silencing profoundly inhibited the proliferation of BMECs compared with controls (Fig. 6a). Further, flow cytometry analysis showed that PTPRF silencing significantly promoted apoptosis (Fig. 6b). In addition, CCK-8 assay demonstrated that PTPRF silencing significantly decreased cell viability compared with the control group (Fig. 6c). Correspondingly, qRT-PCR analysis showed that relative mRNA expression of proliferation marker genes was significantly down-regulated in si-PTPRF-transfected cells compared with si-NC-transfected cells (Fig. 6d), and western blot analysis of PCNA and CDK2 confirmed the qRT-PCR results (Fig. 6e, f).

### PTPRF silencing affects milk protein synthesis by altering mTOR pathway-related gene expression in BMECs

**Figure 7:**
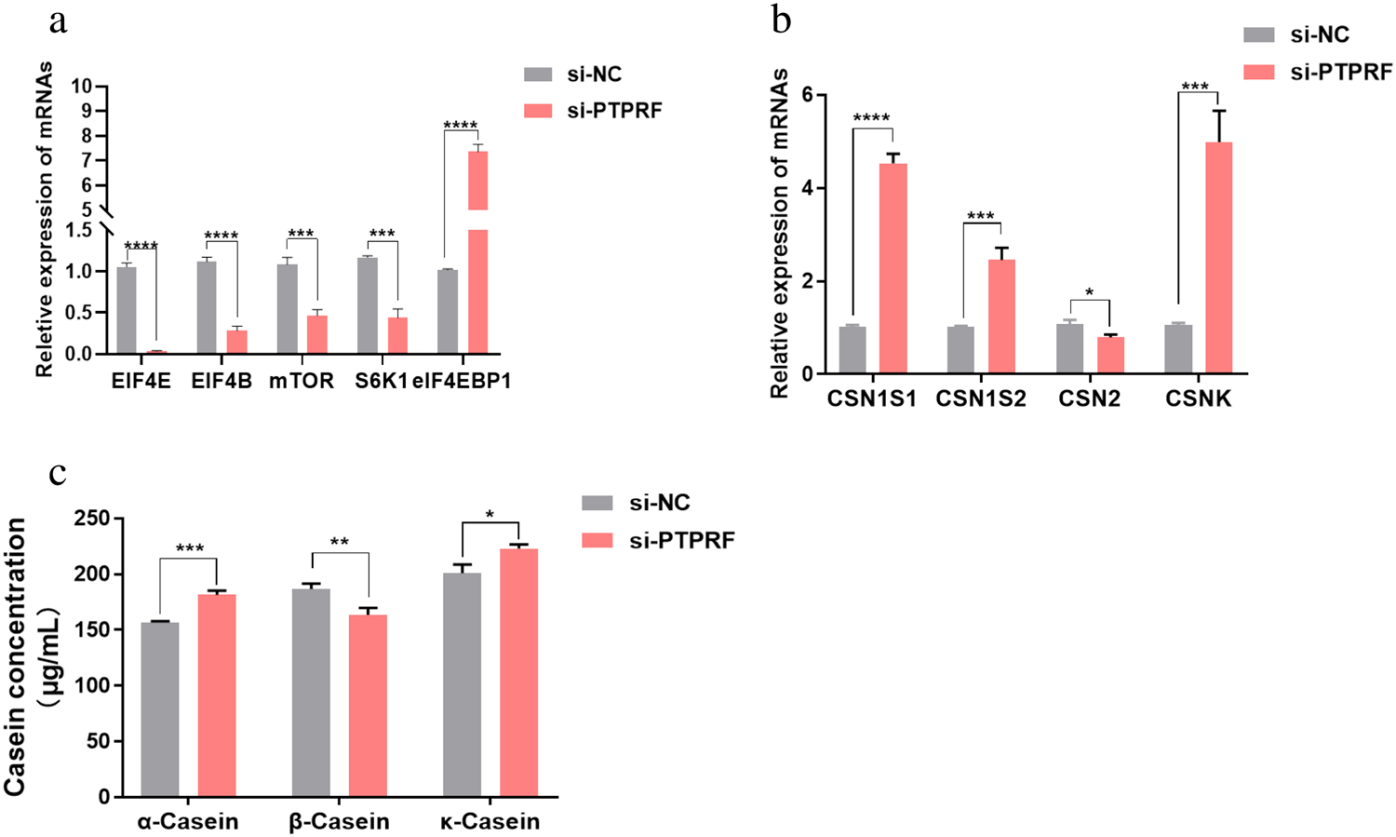
Effect of PTPRF silencing on milk protein synthesis and mTOR signaling in BMECs. (**a**) Relative expression of indicated genes in the mTOR signaling pathway in BMECs transfected with si-PTPRF or si-NC. (**b**) Relative expression of indicated casein-encoding genes in BMECs transfected with si-PTPRF or si-NC. (**c**) Casein concentration in supernatant of BMECs determined by ELISA. *p < 0.05; **p < 0.01; ***p < 0.001; ****p < 0.0001.

We next evaluated the effect of PTPRF silencing on milk protein synthesis and mTOR signaling. We found that the expression of EIF4E, EIF4B, mTOR, and S6K1 were all significantly down-regulated upon PTPRF silencing, whereas eIF4EBP1 expression was significantly up-regulated (Fig. 7a). After transfection with si-PTPRF for 48 h, qRT-PCR analysis showed that PTPRF silencing significantly upregulated CSN1S1, CSN1S2, and CSNK expression and significantly downregulated CSN2 expression compared with controls (Fig. 7b). ELISA analysis of supernatants from the transfected BMECs showed that, compared with si-NC, si-PTPRF significantly increased the amount of α-casein and κ-casein and significantly decreased the amount of β-casein (Fig. 7c); this was consistent with the mRNA expression results and indicates that PTPRF silencing promotes the synthesis of α-casein and κ-casein and reduces β-casein synthesis in BMECs.

## Discussion

Ruminant milk is rich in nutrients and is a part of the daily diet for many people globally. Understanding the regulatory mechanism of miRNA in mammalian physiological processes could provide the theoretical basis for devising strategies to use miRNA to modulate milk quality in animals. The important regulatory role of miRNAs in animal mammary gland development and milk protein metabolism has received increasing attention. For instance, miR-424-5p^9^, miR-2904^10^, miR-139^11^, miR-27a^12^, and miR-8516^13^ have all been shown to regulate milk protein synthesis in mammary epithelial cells. However, most of the studies of miRNA regulation of milk protein synthesis in BMECs have investigated endogenous miRNAs, and there have been few in-depth studies to determine whether plant-derived miRNAs have effects on milk protein synthesis.

There are plenty researches on how to improve the content of milk fat and milk protein in vitro, but the cellular molecular mechanisms are still in the exploratory phase. Recent results suggest that some regulatory networks play a central role in the regulation of the synthesis and secretion processes of dairy cow milk. Among them, JAK-STAT signaling pathway^14^ and mTOR signaling pathway^8^ are the most studied and important pathways on milk protein synthesis. In this study, qRT-PCR was used to detect the expression of mTOR pathway-related genes. Results showed that overexpression of miR159a would increase the expression of eIF4EBP1 gene, while decrease the other mTOR downstream genes expression. This result consist with Wang et.al’s result^15^, Bi’s result^16^, Meng et.al’s result^17^, where the eIF4EBP1 gene has opposite expression with S6K1 and mTOR gene. This result may have occurred because EIF4EBP1 is a translation initiation repressor protein that complexly regulates EIF4E activity, and the negative role of 4EBP1 on protein translation^15,18–20^. In our research, only the mTOR pathway was studied at mRNA level, regulatory role of mTOR pathway at protein level needs to be explored in the future. Also, there are still other possible regulatory networks of dairy cow milk protein synthesis haven’t studied yet and need to be further studied.

Because of its importance in the diet of dairy cows, it is critical to understand whether miRNAs from alfalfa have the potential for cross-kingdom regulatory functions that can affect milk production. In this study, we detected alfalfa xeno-miR159a in the blood and milk of both high-protein-milk and low-protein-milk dairy cows, which demonstrated that xeno-miRNAs could be absorbed into the tissues of dairy cows. This result was consistent with previous studies that demonstrated direct uptake of plant-derived miRNAs into animals through dietary exposure^21–23^. qRT-PCR analysis showed that xeno-miR159a was significantly more abundant in low-protein-milk dairy cows compared with high-protein-milk dairy cows, indicating that xeno-miR159a might regulate milk protein synthesis in dairy cows.

Knowing the target genes of miRNAs is essential to further clarify the signaling pathways they regulate, and the target gene functions of miRNAs regulating milk protein metabolism in breast tissue are mainly involved in milk protein synthesis and secretion. Several target genes related to milk protein metabolism have been shown to be regulated by miRNA. Among them, miR-424-5p targets the 3′UTR of the CSN2 gene and negative regulates the β-casein syntheses^9^, miR-139 inhibits β-casein expression by targeting the growth hormone receptor and insulin-like growth factor receptor in BMECs^11^, and miR-27a negatively regulates milk protein and lactose content by targeting mitogen-activated protein kinase 14 to increase milk protein expression in dairy goats^12^. These studies convincingly demonstrate that miRNAs have important regulatory effects on target genes related to milk protein metabolism in breast tissue; however, research on plant-derived miRNA regulation of mammalian milk production is sparse. Here, we used bioinformatics approaches to determine that alfalfa-derived miR159a can bind the 3′ UTR region of bovine PTPRF, and subsequent functional studies demonstrated that a xeno-miR159a mimic can regulate PTPRF mRNA and protein expression.

**Figure 8:**
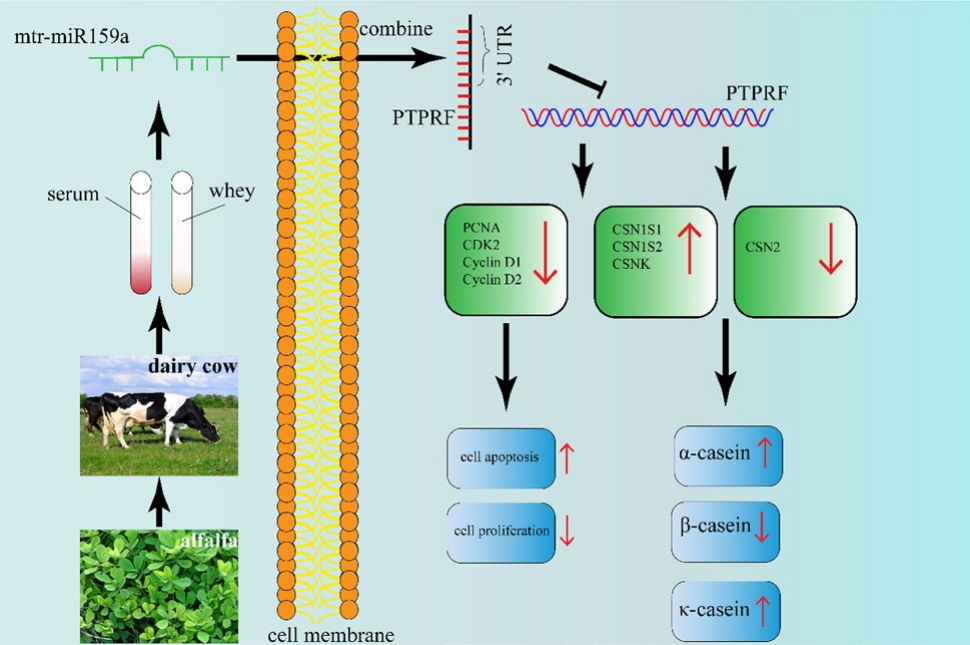
Schematic summary of the mechanism by which alfalfa xeno-miR159a regulates cell proliferation and milk protein metabolism in BMECs.

Plant miR159a has been shown to regulate multiple biological processes of mammals. A synthetic plant-derived miR-159 mimic was able to inhibit breast cancer cell proliferation by targeting the transcription factor TCF7, which is an effector of the Wnt signaling pathway, thereby reducing the level of MYC protein and inhibiting the growth of breast cancer cells^5^. Also, soybean-derived gma-miR159a alleviates colon tumorigenesis by suppressing TCF7/MYC in mice^24^, and exogenous plant gma-miR159a can ameliorate hepatic stellate cell activation and inflammation by inhibiting GSK-3β-mediated pathways, suggesting that gma-miR159a has the potential to prevent liver fibrosis^21^. Despite the potency of plant-derived miR159a, the regulatory role of alfalfa-derived miR159a in milk protein expression remains unknown. We used a chemically synthesized xeno-miR159a mimic in BMECs and found that over-expression of alfalfa xeno-miR159a inhibited BMEC proliferation, promoted apoptosis, increased the concentration of α-casein and κ-casein, and decreased the concentration of β-casein (Fig. 8).

Multiple studies have shown associations between the expression levels of different milk protein components. It was shown that lipoteichoic acid treatment could increase intracellular and secreted αS1-casein, which was accompanied by a decrease in β-casein content in mouse mammary epithelial cells^25^. Also, the levels of α-casein and α-lactalbumin in milk from β-casein and κ-casein non-transgenic cows were higher than in transgenic cows^26^. In our study, over-expression of xeno-miR159a and PTPRF silencing increased the expression of CSN1S1, CSN1S2, and CSNK and decreased the expression of CSN2 at both the mRNA and protein levels, demonstrating that alfalfa xeno-miR159a regulates all three milk protein metabolism genes, also, there may be a negatively correlation between these three casein content. Song^27^ found the content of αS1-casein and β-casein in goat milk showed a negative correlation, and the expression of CSN1S1 and CSN2 genes showed an opposite trend in the mammary glands of goats at lactation stage and middle stage in goat mammary epithelial cell (GMEC), and the amino acid sequences of 10 mammals were analyzed by bioinformatics. It was found that CSN1S1 gene of goats and cattle was most closely related, and the functional modules and functional domains were similar, which may have similar gene functions. This result was similar with our results that β-casein was negatively correlation with α-casein. The reason of this result may be that the interactions and regulatory relationships between different milk protein components are extremely complex, and the regulation mechanism needs to be further studied. In addition, we found that over-expression of alfalfa xeno-miR159a and PTPRF silencing had significant regulatory effects on the expression of mTOR signaling pathway genes, indicating that miR159a may regulate milk protein synthesis through the mTOR pathway; however, further experiments are needed to confirm this hypothesis.

In conclusion, alfalfa xeno-miR159a was detected in the serum and whey of dairy cows, and the abundance of alfalfa xeno-miR159a was significantly higher in cows that produced low-protein milk compared with cows that produced high-protein milk. Xeno-miR159a overexpression inhibited proliferation, promoted apoptosis, and decreased β-casein, while increasing α-casein and κ-casein levels in BMECs. The results of this study provide a foundation for analyzing the effect of alfalfa miRNAs on the molecular regulatory network affecting milk protein synthesis and proliferation of BMECs to improve the nutritional content of milk from ruminant animals. However, our study is an in vitro study which must be validated in vivo further.

## Methods

### Ethics declarations

All experimental protocols were approved by the Ningxia University Technology Ethics Committee, Yinchuan, China (protocol code NXU-23–80). All methods were carried out in accordance with relevant guidelines and regulations. All methods are reported in accordance with ARRIVE guidelines (https://arriveguidelines.org).

### Sample preparation

Milk and blood of 6 Holstein dairy cows (Bos taurus), two alfalfa varieties (Zhongmu No.1 alfalfa and Xinyan alfalfa) from Helanshan Dairy Industry Co., Ltd., in Ningxia Nongken, China, were collected on the same day and analyzed. Milk samples were collected from each cow in the morning, middle and evening of sample collection day for protein quantification, and the 3 cows with higher milk protein (average 3.5 ± 0.54, > 3.3%) were selected as the high-milk-protein group, while the remaining three cows (average 2.7 ± 0.13, < 3.0%) were selected for the low-milk-protein group. High- and low-protein dairy cows were fed the same amount of alfalfa(1.5 kg alfalfa hay and 4 kg alfalfa silage per day). Whole blood was collected by coccygeal venepuncture on the same day when milk samples were collected. Alfalfa, whole blood and fresh milk samples were transported on ice to the lab and stored at − 80 °C until protein and nucleic acid extraction.

Serum separation was conducted by collecting whole blood using 10-ml conical centrifuge tubes without anticoagulant. After collection, samples were left overnight at 4 °C, then centrifuged at 1200 × *g* at 4 °C for 10 min. The supernatants were then transferred to new centrifuge tubes and centrifuged at 1800 × *g* at 4 °C for 10 min, followed by the collection of supernatants and storage at − 80 °C.

Whey separation was conducted by collecting fresh milk samples that were subjected to the following centrifuge protocols: (1) 300 × *g* at 4 °C for 10 min to remove fat, cells, and large debris; (2) 3000 × *g* at 4 °C for 20 min to remove milk proteins; and (3) 10,000 × *g* at 4 °C for 30 min to remove other impurities. Supernatants were then collected and stored at − 80 °C.

### RNA extraction and qRT-PCR analysis

Total RNA was extracted from 5 × 10^5^ cells/mL transfected BMECs in 6-well plates, alfalfa and from bovine serum and whey by the Trizol method according to the manufacturer’s instructions (Takara, Kyoto, Japan). The concentration (ng/µL) and OD (260/280) of total RNA were measured by a multifunctional full-wavelength microplate reader, and the integrity of total RNA was assessed by 1% agarose gel. To generate cDNA, 1000 ng of the obtained total RNA was reverse transcribed using random primers provided in a reverse transcription kit (Takara, Kyoto, Japan), and cDNA was then stored at − 20 ^ο^C. qRT-PCR was performed using 2 × chamq universal SYBR qPCR master mix (Vazyme, Nanjing, China), on cDNA templates extracted from BMECs, alfalfa, and bovine serum or whey; three parallel replicates were performed on each biological sample. Primer Premier 5.0 (Premier Biosoft, CA, USA) software was used to design qRT-PCR primers (Table S1). The qRT-PCR reaction was carried out in a 20 μL PCR mix consisting of 10 μL of ChamQ SYBR qRT-PCR Master Mix, 0.4 μL each of 100 μM forward and reverse primers, and 2.0 μL cDNA (100 ng/μL). The PCR reaction conditions were as follows: for CSN2, pre-denaturation at 95 °C for 30 s followed by 44 cycles of 95 °C for 10 s and 51.7 °C for 30 s; and for all other genes, pre-denaturation at 95 °C for 30 s followed by 40 cycles of 95 °C for 5 s and 60 °C for 30 s. GAPDH and 18S rRNA were used as internal references.

### Oxidation experiment

10 μL of the extracted RNA sample was mixed with 90 μL 10 mM sodium periodate and placed in -20℃ refrigerator for oxidation reaction for 40 min. The precipitate was resuspended with 1 mL of absolute ethanol and placed in 4℃ refrigerator for 30 min. Then, the mixture was centrifuged at 12,000 × g at 4℃ for 15 min, after which the supernatant was discarded. After centrifuging, 1 mL of 75% absolute ethanol was added to resuspend the precipitate again, which was placed in a 4℃ refrigerator for 15 min, and then, the mixture was centrifuged at 12,000 × g at 4℃ for 15 min again. After absolute ethanol was discarded, 20 μL of enzyme-free water was added to dissolve the RNA precipitate^2^. The alfalfa-derived mtr-miR159a and bovine-derived bta-miR-16a in the oxidized RNA solution were quantified using qRT-PCR and the plant-derived miRNAs were verified.

### Cell culture and transfection

BMECs and human kidney epithelial cells (HEK-293 T) were obtained from Ningxia University, Key Laboratory of Ruminant Molecular Cell Breeding of Ningxia Hui Autonomous Region. BMECs were cultured with DMEM/F12 (Hyclone, UT, USA) cell media containing 10% FBS (BI, Beit Haemek, Israel), penicillin (100 IU/mL), streptomycin (100 μg/mL), hydrocortisone (5 μg/mL), insulin (5 μg/mL) and prolactin (20 ng/mL) at 37 °C with 5% CO2 concentration and 100% humidity. HEK-293 T cells were cultured in high-sugar DMEM (Hyclone, UT, USA) with 10% FBS (BI, Beit Haemek, Israel) at 37 °C, 5% CO2 modified atmosphere, and 100% humidity. All cells used in this study were taken from the third passage after resuscitation.

The sequence used for the xeno-miR159a mimic was 5′-TTTGGATTGAAGGGAGCTCTA-3′. The sequences for si-PTPRF were: sense, 5′-GCGACAGCAAACCUGUCUUTT-3′ and antisense, 5′-AAGACAGGUUUGCUGUCGCTT-3′. miRNA mimic and a miRNA negative control (NC; hereafter, NC mimic) were purchased from RiboBio Co., Ltd. (RiboBio Co., Ltd, Guangzhou, China), si-PTPRF and si-NC were purchased from Sangon Biotech Co., Ltd. (Sangon Biotech Co., Ltd, Shanghai, China). Cells were seeded at 3×10^5^ cells/ml in 6-well plates and allowed to grow until approximately 60% confluency to transfected with 45 nM xeno-miR159a mimic, 45 nM NC mimic, 80 nM si-PTPRF, or 80 nM si-NC using lipofectamine 3000 (Invitrogen, USA) transfection reagent for 48 h at 37 °C. Cells were used for studies after 48 h of transfection.

### CCK-8 assay

BMECs were inoculated and cultured in 96-well plates. When cell density reached 60–70%, they were transfected with xeno-miR159a mimic, NC mimic, si-PTPRF, or si-NC. After 48 h, 10 μL CCK-8 enhanced solution (Meilunbio, Dalian, China) was added, and cell viability was calculated by measuring OD at 450 nm after a 1-h incubation; three biological and technical replicates in parallel of each treatment were measured. Cell viability was calculated by the following formula:

$${\text{Cell viability}}\, = \,[({\text{As}} - {\text{Ab}})/({\text{Ac}} - {\text{Ab}})]\, \times \,{1}00\%$$

As: Experimental group, Ab: control group, Ac: blank group.

### 5-Ethynyl-2′-deoxyuridine (EdU) staining

BMECs were inoculated and cultured in 6-well plates. When cell density reached 60-70%, xeno-miR159a mimic, NC mimic, si-PTPRF, or si-NC were transfected; after 48h, proliferation of the BMECs was measured using the BeyoClick EdU-555 Cell Proliferation Kit (Beyotime, Shanghai, China) according to the manufacturer’s protocol. Treated cells were observed and photographed with a fluorescence microscope (Olympus Corporation, Japan). The cytoplasm of newly proliferated cells was identified by red fluorescence, and the nuclei of all cells were identified by blue fluorescence; three biological and technical replicates in parallel of each treatment were measured.

### Apoptosis analysis

Cell apoptosis was detected by flow cytometry using the Annexin V-FITC apoptosis detection kit (Beyotime, Shanghai, China) according to the manufacturer’s protocol. To detect apoptosis, the cell culture medium was transferred into centrifuge tubes, the adherent cells were washed using PBS, then, trypsin cell digestion solution was added. The digestion was stopped by adding cell culture medium after 5 min. Then, cells were centrifuged at 1000 × *g* for 5 min, the supernatant was discarded, and cells were re-suspended using PBS and heated in a 50 ^°^C water bath for 2 to 3 min and then subjected to apoptosis stimulation. Next, re-suspended cells were centrifuged at 1,000×g for 5 min; the supernatant was discarded and the cells were re-suspended gently in 195 µL AnnexinV–FITC conjugate and then 5 µl AnnexinV–FITC was added. Next, 10 µL propidium iodide staining solution was added. After incubation in the dark at room temperature (25 ^ο^C) for 25 min, the apoptotic cells were detected by C6 flow cytometer^28^. Three biological and technical replicates in parallel of each treatment were measured.

### ELISA

Following transfection and subsequent incubation for 48 h, the supernatants of BMEC cultures from each treatment group were collected, and the secretion of α-casein, β-casein, and κ-casein was detected using the relevant ELISA kits (CZi Bio CO., Ltd., Shanghai, China) according to the manufacturer’s instructions. Three parallel replicates of each treatment were measured.

### Construction of recombinant plasmid and dual-luciferase reporter gene assays

The target gene of xeno-miR159a was predicted using the TargetScan database (https://www.targetscan.org/). The restriction endonucleases XhoI and NotI were used to construct a wild-type 3’UTR-wt-CHECK-2 double luciferase reporter gene vector. To verify the binding site of the xeno-miR159a on PTPRF, we commissioned Sangon Biotech Co., Ltd. (Shanghai, China) to chemically synthesize a vector with mutations (mut) at the potential binding site of PTPRF and then constructed 3’UTR-mut-CHECK-2 mutant vectors. All constructs were verified by Sanger sequencing.

Human 293T cells are usually selected for dual-luciferase reporter gene assays due to its higher transfection efficiency, also, the protein expressed by 293T cells has high repeatability and consistency among batches, and transfection with 293T cells can also eliminate the interference of some endogenous genes^17^. Human 293T cells were inoculated and cultured in 24-well plates. When cell density reached 60-70%, the 3’UTR-wt-CHECK-2 (or 3’UTR-mut-CHECK-2) recombinant plasmid was co-transfected with xeno-miR159a mimic into 293T cells using lipofectamine 3000 (Invitrogen, USA) transfection reagent or with recombinant plasmid and NC mimic. Luciferase activity was analyzed using the Dual-Luciferase Reporter Assay System according to the manufacturer’s instructions (Promega, WI, USA). Three biological and technical replicates in parallel of each treatment were measured.

### Western blot

Total protein was extracted from BMECs using a whole-protein extraction kit (KeyGEN, Nanjing, China), and protein concentration was determined using a BCA protein assay kit (KeyGEN, Nanjing, China) according to manufacturer’s instructions. A total of 100 μg protein was loaded onto 10% SDS polyacrylamide gels, separated by electrophoresis, and transferred onto polyvinylidene difluoride (PVDF) membranes. The membranes were blocked in a protein free rapid blocking buffer (Epizyme Biotech, Shanghai, China) at room temperature for 15 min and the membranes were then incubated with antibodies targeting proteins of interest. Primary antibodies were used to detect GAPDH (AB0036, 1:3000, Abways), PTPRF (A5444, 1:2000, ABclonal), PCNA (D220014, 1:500, Sangon Biotech) and CDK2 (1:500, Sangon Biotech), and goat anti-rabbit IgG (ZB-2301, 1:20,000, ZSGB-Bio) was used as the secondary antibody. A chemiluminescent ECL Western blot system (Tanon-5200, China) was used for signal detection, and protein was quantified using Image J software. Three biological and technical replicates in parallel of each treatment were measured.

### Data analysis

At least three biological replicates and three technical replicates were performed for each experiment. Relative gene expression was calculated using the 2-ΔΔct formula. The two-tailed and unpaired Student’s t-test were used to compare two groups and one-way ANOVA was for more than two groups using GraphPad Prism8 software (GraphPad Software, Inc., La Jolla, CA, USA), with p < 0.05 set as the level for statistical significance (*), and p < 0.01 set as the level for extreme statistical significance (**).

### Supplementary Information

Supplementary Information.

## Data Availability

The datasets analyzed during the current study are available in the NCBI repository, [http://www.ncbi.nlm.nih.gov/bioproject/822492].
