# Supplementary material for: Alfalfa xeno-miR159a regulates bovine mammary epithelial cell proliferation and milk protein synthesis by targeting PTPRF
Source: Sci Rep. 2024 Apr 20;14:9117. doi: 10.1038/s41598-024-59948-x (PMC11032358; doi:10.1038/s41598-024-59948-x)
Supplement: Supplementary file 1 — Supplementary Information. [file 41598_2024_59948_MOESM1_ESM.pdf]

Supplementary Information

Alfalfa xeno-miR159a Regulates Bovine Mammary Epithelial Cell Proliferation and Milk Protein Synthesis by Targeting PTPRF

Hongjuan Duan[1], Shaojin Li[1], Chuangwei Li[1], Lan Tian[1], Yun Ma[1], Xiaoyan Cai[1,*]

Table S1 Information for qRT-PCR.

gene	sequence（5'-3'）	length of product（bp）
PTPRF	F：CAACACAAGTGCCAAGCTGT<br>R：AGGGTCCACAGGAAGGAAGT	184
CSN2	F：GAGGAATCTATTACACGCATCA<br>R：TTTGTGGGAGGCTGTTAT	160
CSNK	F：GCCCAGGAGCAAAACCAAGA<br>R：GGACTTGGCAGGCACAGTAT	264
CSN1S1	F：ATGTGCCCTCTGAGCGTTAC<br>R：AGGCACCAGATGGATAGGC	237
CSN1S2	F：AATCAGCCAGCGTTACCAG<br>R：CCAGTCCAACCATAACCAGG	170
mTOR	F：AAAGGCATGTTCGAGGTGCT<br>R：GCTGCTTGGAGATTCGTCTG	211
eIF4EBP1	F：CACTAGCCCTACAGGCGAT<br>R：GCTGGTGTCCACGAAGAAGA	298
eIF4E	F：AACGAGGAGGACGATGGCTA<br>R：AGCCGCTCTTAGTAGCTGTG	303
eIF4B	F：GTAGAAGAGCGGCTACAGA<br>R：GTTCCCGTTCCTGAGTTT	124
S6K1	F：CACCTGTTGACAGCCCAGAT<br>R：CGAGGGGATCGGATTTTTGG	139
GAPDH	F：GGCATCGTGGAGGGACTTATG<br>R：GCCAGTGAGCTTCCCGTTGAG	186
CDK2	F：ATGAACTGACCAGGAGGG<br>R：GCCAGGAGTTACTTCTATGC	115
Cyclin D1	F：CATGAACTACCTGGACCGCT<br>R：TCTTGGAGAGGAAGTGCTCG	260

Cyclin D2	F：CACCGATGTGGATTGCCTCA<br>R：TCCAGCTCATCCTCCGACTT	117
PCNA	F: TCCAGAACAAGAGTATAGC<br>R: TACAACAGCATCTCCAAT	94
mtr-miR162	RT:GTCGTATCCAGTGCAGGGTCCGAGGT<br>ATTCGCACTGGATACGACCTGGAT<br>F: TCCGGTCGATAAACCTCTGC<br>R: GGGTCCGAGGTATTCGCACT	55
mtr-miR2643a	RT: GTCGTATCCAGTGCAGGGTCCG<br>AGGTATTCGCACTGGATACGACTCTCTAAT<br>F: CGTTGATTTGGGATCAGAA<br>R: CAGTGCAGGGTCCGAGGTAT	63
mtr-miR396a	RT:GTCGTATCCAGTGCAGGGTCCGAG<br>GTATTCGCACTGGATACGACAAGTTCAA<br>F: CGCGTTTTCCACAGCTTTC<br>R: AGTGCAGGGTCCGAGGTATT	62
mtr-miR166a	RT:GTCGTATCCAGTGCAGGGTCCGAGGT<br>ATTCGCACTGGATACGACTGCACGTG<br>F: CGCAGACAGTTGGAGAAGCAGGG<br>R: AGTGCAGGGTCCGAGGTATTCGCAC	54
bta-miR-16a	RT: CTCAACTGGTGTCGTGGAGTCGGCAA<br>TTCAGTTGAGCACCAA<br>F: GCCCGTAGCAGCACGTAAAT<br>R: TGTCGTGGAGTCGGCAAT	54
mtr-miR159a	RT: GTCGTATCCAGTGCAGGGTCCGAGGTAT<br>TCGCACTGGATACGACTAGAGCTC<br>F: CGGCTTTGGATTGAAGG<br>R: TGCAGGGTCCGAGGTATT	80
18S	F: TTTCGATGGTAGTCGCTGTG<br>R: GGATGTGGTAGCCGTTTCT	98

Table S2. miRNA sequences and expression in alfalfa.

miRNA	Sequence (5′-3′)	Expression in Zhongmu No.1 alfalfa	Expression in Xinyan alfalfa
mtr-miR159a	TTTGGATTGAAGGGAGCTCTA	180,765.61	202,441.59
mtr-miR396a	TTCCACAGCTTTCTTGAACTG	94,603.75	105,374.70
mtr-miR166a	TCTCGGACCAGGCTTCATTCC	36,077.26	27,206.53
mtr-miR2643a	TTTGGGATCAGAAATTAGAGA	6,881.68	10,035.44

mtr-miR162	TCGATAAACCTCTGCATCCAG	3,154.56	6,165.89

Table S3. 260/280 readings of extracted miRNA.

name	260	280	260/280
xeno-miR159a mimic 1	1.643	0.832	1.974
xeno-miR159a mimic 2	1.593	0.811	1.966
xeno-miR159a mimic 3	0.752	0.395	1.903
NC mimic 1	1.175	0.606	1.941
NC mimic 2	1.388	0.719	1.931
NC mimic 3	0.844	0.464	1.82

Figure S1. images of the agarose gels used to check RNA integrity.

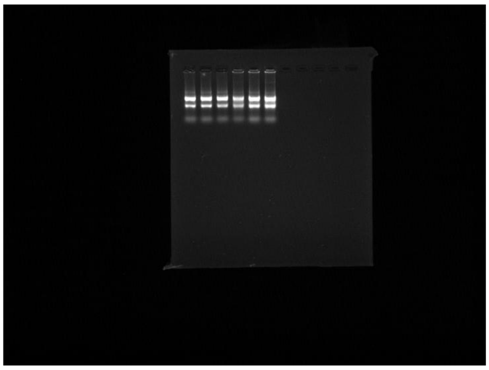

Figure S2 Original blots of Western Blot

A. Western Blot for CDK2(34 kDa)

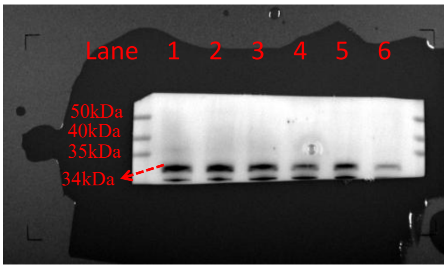

Lane 1,2,3 NC mimic

Lane 4,5,6 xeno-miR159a mimic

(for Figure 3)

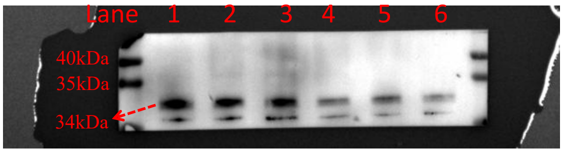

Lane 1,2,3 si-NC

Lane 4,5,6 si-PTPRF

(for Figure 6)

B. Western Blot for PCNA(36 kDa)

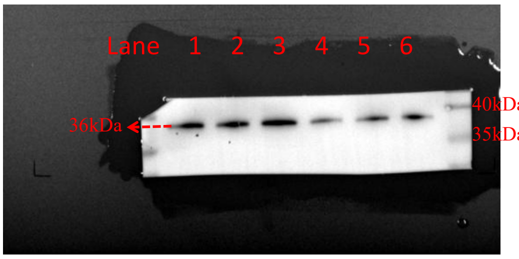

Lane 1,2,3 NC mimic

Lane 4,5,6 xeno-miR159a mimic

(for Figure 3)

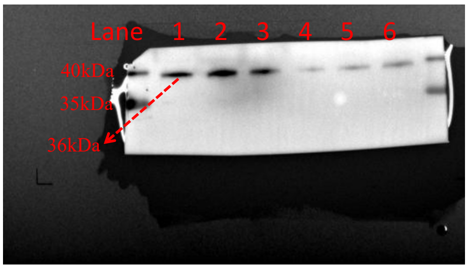

Lane 1,2,3 si-NC

Lane 4,5,6 si-PTPRF

(for Figure 6)

C. Western Blot for PTPRF(150 kDa)

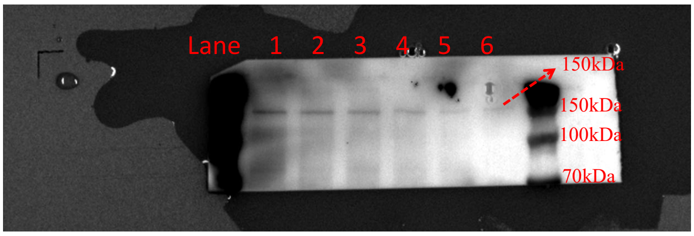

Lane 1,2,3 NC mimic

Lane 4,5,6 xeno-miR159a mimic

(for Figure 5)

D. Western Blot for GAPDH(36 kDa)

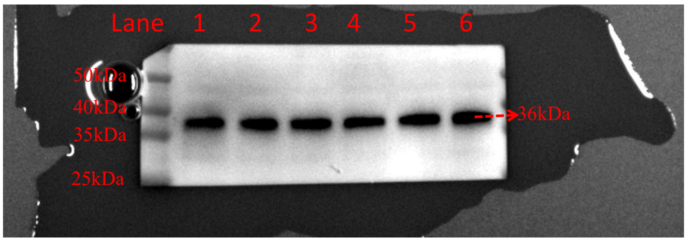

Lane 1,2,3 si-NC

Lane 4,5,6 si-PTPRF

(for Figure 6)

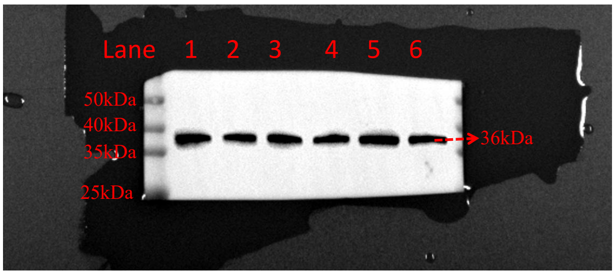

Lane 1,2,3 NC mimic

Lane 4,5,6 xeno-miR159a mimic

(for Figure 3)

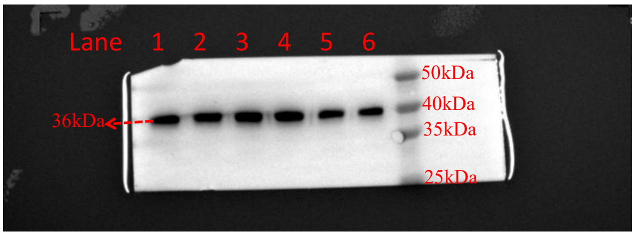

Lane 1,2,3 NC mimic

Lane 4,5,6 xeno-miR159a mimic

(for Figure 5)
